# The effect of ultrasound pulse length on microbubble cavitation induced antibody accumulation and distribution in a mouse model of breast cancer

**DOI:** 10.7150/ntno.46892

**Published:** 2020-09-15

**Authors:** Marie Amate, Joseph Goldgewicht, Balasubramanian Sellamuthu, John Stagg, Francois T.H. Yu

**Affiliations:** 1 Microbubble Theranostic Laboratory, CHUM Research Center, Montreal, Canada.; 2 Department of Radiology, Radiation Oncology and Nuclear Medicine, Faculty of Medicine, Université de Montréal.; 3 Faculty of Pharmacy, Université de Montréal.

**Keywords:** antibody therapy, ultrasound, microbubbles, image guided therapy, solid cancer, immune check-point blockade, extravasation.

## Abstract

In solid tumors, the limited diffusion of therapeutic molecules in the perivascular space is a known limitation impacting treatment efficacy. Ultrasound Targeted Microbubble Cavitation (UTMC) has been shown to increase vascular permeability and improve the delivery of therapeutic compounds including small molecules, antibodies (mAb), nanoparticles and even cells, notably across the blood-brain-barrier (BBB). In this study, we hypothesized that UTMC could improve the accumulation and biodistribution of mAb targeting the adenosinergic pathway (i.e. CD73) in mice bearing bilateral subcutaneous 4T1 mammary carcinoma. **METHODS:** A bolus of fluorescently labeled mAb was given intravenously, followed by a slow infusion of microbubbles. UTMC therapy (1 MHz, 850 kPa) was given under ultrasound image guidance for 5 minutes to the right side tumor only, using three different pulse lengths with identical ultrasound energy (5000cyc “long”, 125x40cyc “mid” and 500x10cyc “short”), and leaving the left tumor as a paired control. Longitudinal accumulation at 0 h, 4 h and 24 h was measured using whole-body biofluorescence and confocal microscopy. **RESULTS:** Our data support an increase in antibody accumulation and extravasation (# extravasated vessels and extravasated signal intensity) at 0 h for all pulses and at 4 h for the mid and short pulses when compared to the control non treated side. However, this difference was not found at 24 h post UTMC, indicative of the transient nature of UTMC. Interestingly, confocal data supported that the highest extravasation range was obtained at 0 h with the long pulse and that the short pulse caused no increase in the extravasation range. Overall, the mid pulse was the only pulse to increase all our metrics (biofluorescence, fraction of extravasated vessels, amount of extravasated Ab, and extravasation range) at 0 h and 4 h time points. **CONCLUSIONS:** Our results support that UTMC can enhance antibody accumulation in solid tumors at the macroscopic and microscopic levels. This preferential accumulation was evident at early time points (0 h and 4 h) but had started to fade by 24 h, a time dependence that is consistent with the ultrasound blood brain barrier opening literature. Further development and optimization of this theranostic platform, such as repeated UTMC, could help improve antibody based therapies against solid cancer.

## Introduction

Monoclonal antibodies (mAbs) are widely used in clinical cancer care and continue to play an increasingly large role in drug development by specifically targeting cancer cells, immune cells or the vasculature 1. mAbs can be used in their native form, altered so to increase or suppress immune activation or conjugated with a cytotoxic agent for targeted delivery 2. A new class of mAbs targets so-called immune checkpoint pathways (e.g. PD-1/PD-L1 and CTLA-4) in order to stimulate anti-tumor immune responses 3. They operate by overcoming immune suppressive pathways thereby unleashing immune-mediated killing of tumor cells. mAbs targeting PD-1/PD-L1, for instance, are associated with impressive response rates, reaching ~30% across many cancer types. In addition to PD-1 and CTLA-4, numerous other immune checkpoints have been shown to stimulate anti-tumor immunity. Amongst the next generation of immune checkpoint inhibitors are mAbs targeting the CD73 ecto-nucleotidases responsible for the generation of extracellular and immunosuppressive adenosine 4, 5.

While mAbs can be highly effective anti-cancer drugs, they are bulky molecules that do not distribute evenly in solid tumors, where high interstitial pressure and chaotic vasculature further impedes the normal physiological interstitial perfusion from the blood stream to cells in tissues 6-8. This inhomogeneous distribution limits the efficacy in antibody based therapy. Interestingly, microbubbles (MB) stimulated by a spatially targeted ultrasound (US) field has been shown to cause several bioeffects that could enhance antibody delivery and favor immunotherapy in solid tumors. For instance, it is well known that MB oscillations can increase vascular permeability, notably in the brain, causing a transient opening of the blood brain barrier (BBB). This technology was shown to be safe in a first in-human study concluded recently 9 and to increase the delivery of many compounds to the brain, ranging from small molecular drugs (< 1 kDa) (e.g. doxorubicin) and dyes (e.g. gadolinium or Evan's blue) to antibodies (100 kDa) (e.g. Herceptin), nanoparticles (20-50 nm), and even cells (several μm) 10-13. While the benefits of vascular escape are evident for drug delivery in the context of the BBB, it remains unknown if increasing vascular permeability would be beneficial for tumoral drug delivery, in which the leaky vasculature is already known to support the enhanced permeability and retention effect. Interestingly, UTMC has been shown to increase the extravasation of 60 kDa FITC dextran in xenografted mouse CT26 tumor model 14. Recently, using dorsal window chambers and two-photon microscopy, it was shown that UTMC dramatically increased the extravasation of 2 MDa FITC dextrans and nanoparticles in a human osteosarcoma model 15. Nevertheless, much remains to be understood about the effect of MBs and ultrasound on antibody accumulation in solid tumors, including its spatial distribution and temporal kinetics. A recent study reported that combining UTMC with antibody check-point blockade (anti-PD-1) induced tumor growth inhibition and an increase in survival in the treated group 16, however the mechanism remains unclear, since there was no evidence of an increase in tumor infiltrating lymphocytes. In another study, UTMC has been shown to increase the efficacy of cetuximab (anti-EGFR mAb) in a squamous cell carcinoma syngeneic mouse model 17. The authors reported an increase in cellular mAb uptake following UTMC *in vitro* but the *in vivo* tumoral antibody distribution was not explored. The ultrasound therapeutic pulsing scheme differed significantly between these studies in terms of pressure, total number of cycles, treatment duration and most importantly pulse length. Consequently, there is a need to better understand the effects of UTMC on antibody accumulation and tumoral distribution and to explore the ultrasound parameter space that can help improve UTMC + antibody immunotherapy.

In tumors, extracellular ATP is released in response to cellular stress 18 and gets degraded into adenosine by ecto-nucleotidases, notably by CD39 (hydrolyzing ATP to AMP) and CD73 (hydrolyzing AMP to adenosine). Adenosine drives potent immunosuppressive signals on virtually all major immune cells 19 and are foreseen as the next generation of immune checkpoint inhibitors 5, 20. CD73 is overexpressed in many cancers 19 and anti-CD73 mAb therapy has been shown to block conversion of AMP into adenosine and to enhance the activity of anti-PD-1 or anti-CTLA-4 immune checkpoint inhibitors 20, 21.

In this study, we hypothesized that UTMC would enhance anti-CD73 mAb accumulation and tumoral distribution in the 4T1 murine mammary carcinoma model. This hypothesis was tested using *in vitro* and *in vivo* experiments using whole body biofluorescence imaging and confocal microscopy.

## Methods

### Bilateral tumor model and experimental protocol

All animal protocols used in this study were approved by the CHUM research center Institutional Animal Protection Committee. The CHUM Research Center holds a Certificate of Good Animal Practice from the Canadian Council on Animal Care (CCAC). Mammary carcinoma tumor cells (4T1), kindly provided by Dr Stagg (CRCHUM), were cultured in RPMI 1640 medium, supplemented with 10% fetal bovine serum (FBS) and 1% penicillin-streptomycin and passaged before reaching confluence (<80%). 4T1 cells (1×10^6^) were mixed (1:1vol) with Matrigel matrix (Corning, Tewksbury, MA) and implanted subcutaneously and bilaterally into 8-10 weeks old female BALB/c mice (Charles Rivers) in the posterior upper flank area (100 μL/side).

**Figure 1:**
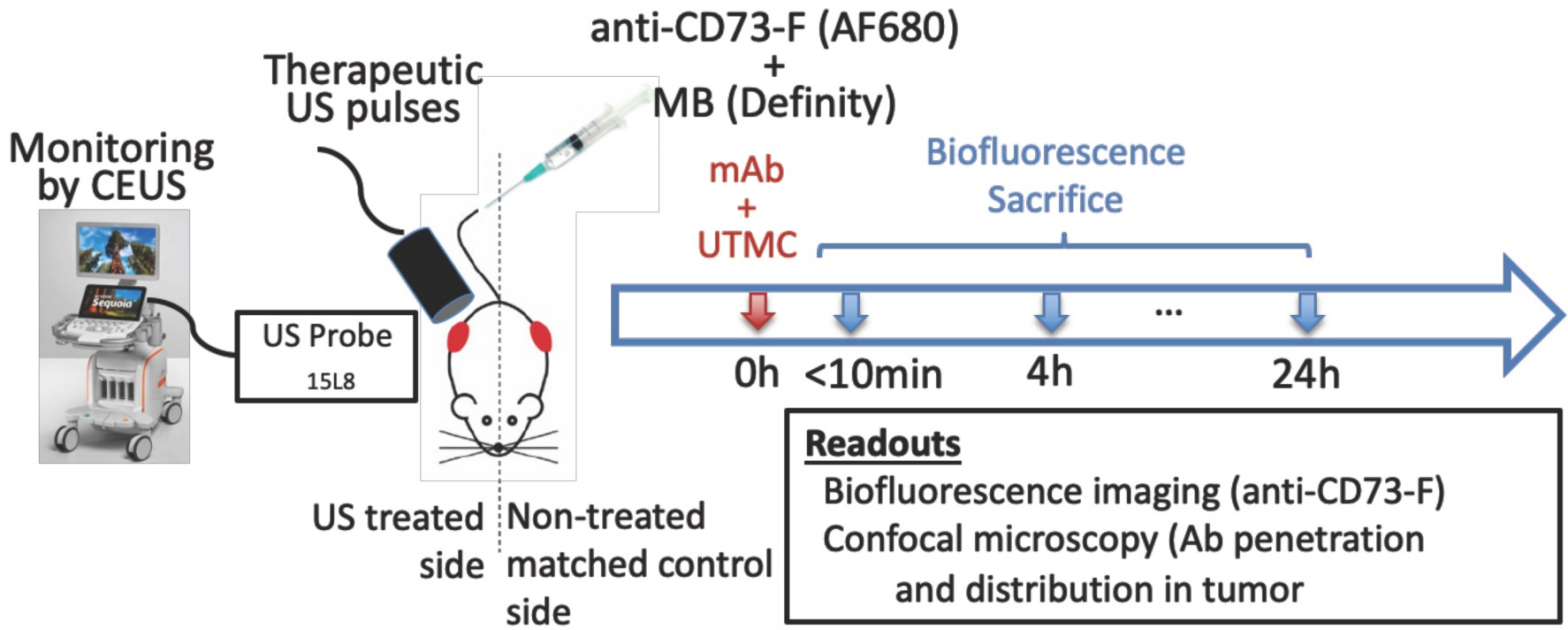
** Experimental approach:** a) Mice bearing bilateral subcutaneous tumors (volume of ~250 mm^3^ each) received a systemic bolus (100 μg) of fluorescently labeled antibodies (anti-CD73-F) and ultrasound targeted microbubble cavitation (UTMC) therapy on the right side during the infusion of microbubbles (Definity) via the tail vein. UTMC was guided by contrast enhanced ultrasound imaging (CEUS) allowing monitoring of the spatially targeted therapy and microbubble replenishment. Mice were then imaged for biofluorescence and sacrificed at 0 h (<10min), 4 h and 24 h post UTMC for confocal microscopy.

Tumor size was measured using calipers every 2-3 days. Volume was calculated as (length × width × depth / 2). When the tumors reached 200-300 mm^3^ (between days 12 and 17 post tumor cell implantation, see Figure 5), mice were anesthetized using 1.5% isoflurane, the tumoral areas were depilated and a bolus (100 μg) of anti-CD73 antibodies (TY/23, Bioxcell, West Lebanon, NH), previously fluorescently labeled (anti-CD73**-F)** using a commercial kit (AlexaFluor680, SAIVI rapid antibody labeling kit, Invitrogen) and counted using a nanodrop system (Nanodrop, Thermo Scientific), was injected intravenously. Antibody injection time was the reference time (t=0 h) for the experiment. This was followed by a slow infusion (4 μL/min) of MBs (Definity, Lantheus) via the tail vein. Microbubbles were activated using a Vialmix amalgamator as specified by the manufacturer. The syringe was constantly rotated to avoid MB floatation. Therapeutic US was delivered on the right tumor for 5 minutes using a single element commercial unfocussed transducer (1 MHz, 0.5 inch, A303S, Olympus), under live guidance using contrast enhanced US (CEUS) imaging (CPS7, Sequoia, Siemens), whereas the left tumor served as a paired control. Mice were then imaged by near infrared optical fluorescence imaging (670 nm/700 nm [Ex/Em], OptixMX2, GE Healthcare, Chicago IL) within 5 min post therapy. The biofluorescence imaging was thus performed within 10 min post mAb injection (labeled “0 h” thereafter for simplicity), and at 4 h and 24 h. Mice received an injection of vascular stain (Hoechst33342, 20 μg/g, Invitrogen) 10 minutes before sacrifice by perfusion fixation through the heart. Tumors were collected and immediately snap frozen in O.C.T. compound (Fisher Healthcare Tissue-Plus, Fisher Scientific company, Ottawa, Canada) for confocal microscopy imaging (Figure 1). A total of 27 mice were used in this study, 3 mice per group x 3-time points x 3 ultrasound pulses.

### Ultrasound Image-Guided Therapy

Therapeutic US consisted of 1 MHz, 850 kPa, 5000 cycle pulses given every 5 s for 5 minutes, either as a continuous train of 5000 cycles (“long” pulse) or subdivided 125 trains of 40 cycles (“mid” pulse) or into 500 trains of 10 cycles (“short” pulse), spaced by 100 μs, such that the total number of acoustic cycles per pulse (5000 cycles) and acoustic energy were the same in all three groups. The therapeutic transducer was driven by an arbitrary function generator (33210A, Keysight technologies, Santa Rosa, CA) connected to a power amplifier (AR, model 75A250, Souderton, PA, USA) and was previously fully calibrated using a membrane hydrophone (HMA0200, Onda Corporation, CA) in a degassed water tank equipped with a 3D motor positioning system. The chosen pressure was the highest pressure that could be delivered by our amplifier. The therapeutic transducer was positioned on top of the right tumor, perpendicularly to the imaging probe plane such that the treatment pulse could be clearly localized in CEUS imaging, thus providing validation of the probe alignment and real-time therapeutic delivery monitoring as well as confirmation of MB replenishment between UTMC pulses (Figure 1). An acoustic absorbing pad was placed under the animal to prevent standing waves from developing.

### Biofluorescence Imaging and image analysis

Antibody accumulation at 0 h (less than 10 min after therapy) (N=9), 4 h (N=6) and 24 h (N=3) was quantified by biofluorescence using a whole body animal bio-imager (OptixMX2, GE). Mice were maintained under anesthesia by 1.5% isoflurane and scanned with all imaging parameters fixed for all animals (laser, filter, resolution, integration time). Tumors were manually delimited and the mean intensity was calculated using the manufacturer's dedicated software (Optiview, GE Healthcare, Chicago, IL). The paired design ensured a tight control on the measured signals since the only difference between both sides was the application of the ultrasound therapy on the treated side.

### Confocal imaging and signal processing

**Figure 2:**
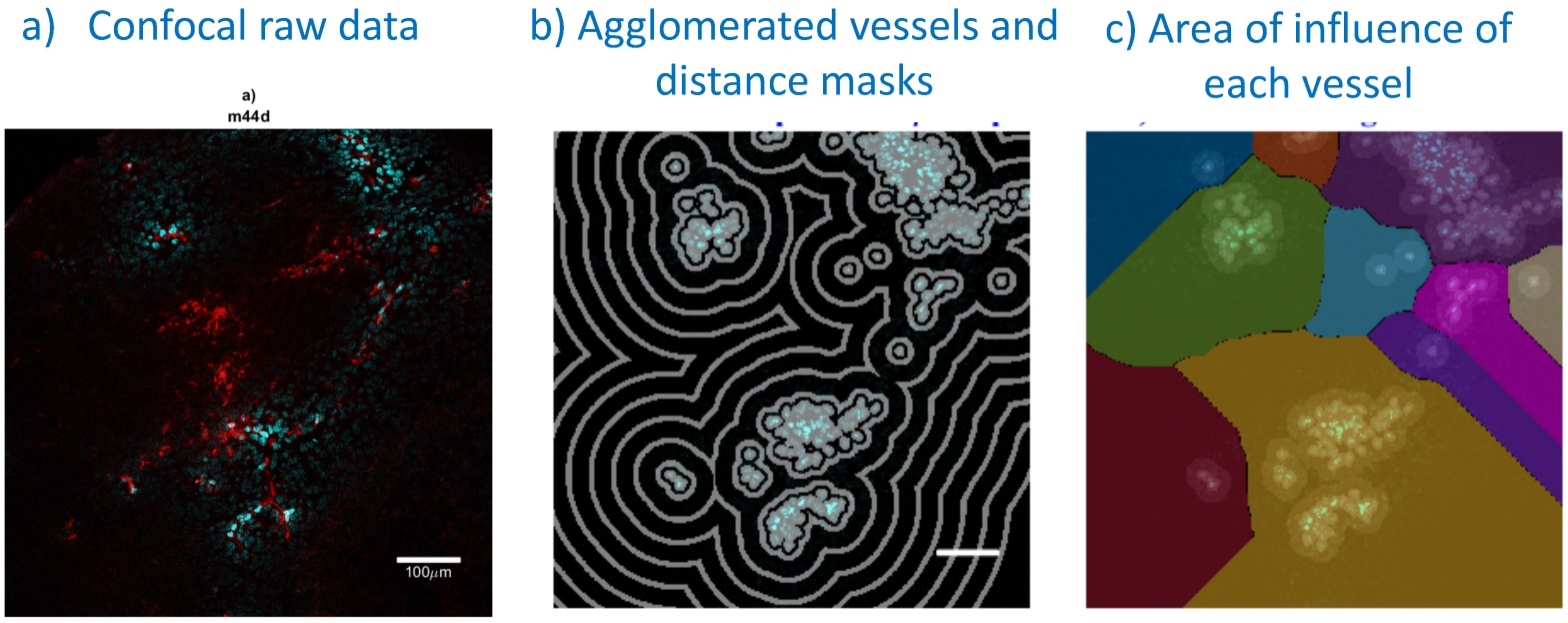
** Confocal image analysis, including vessel segmentation and determination of each vessel area of influence**: a) A typical example of a confocal image (treated side, 5000cyc, 0 h): perfusion stain (Hoechst33342) in blue and antibody fluoresence in red; b) vessel agglomeration and distance masks (spaced 36 µm apart) used in distance analyses; c) watershedding operator to determine the area of influence of each vessel. Bar is 100 μm.

**Figure 3:**
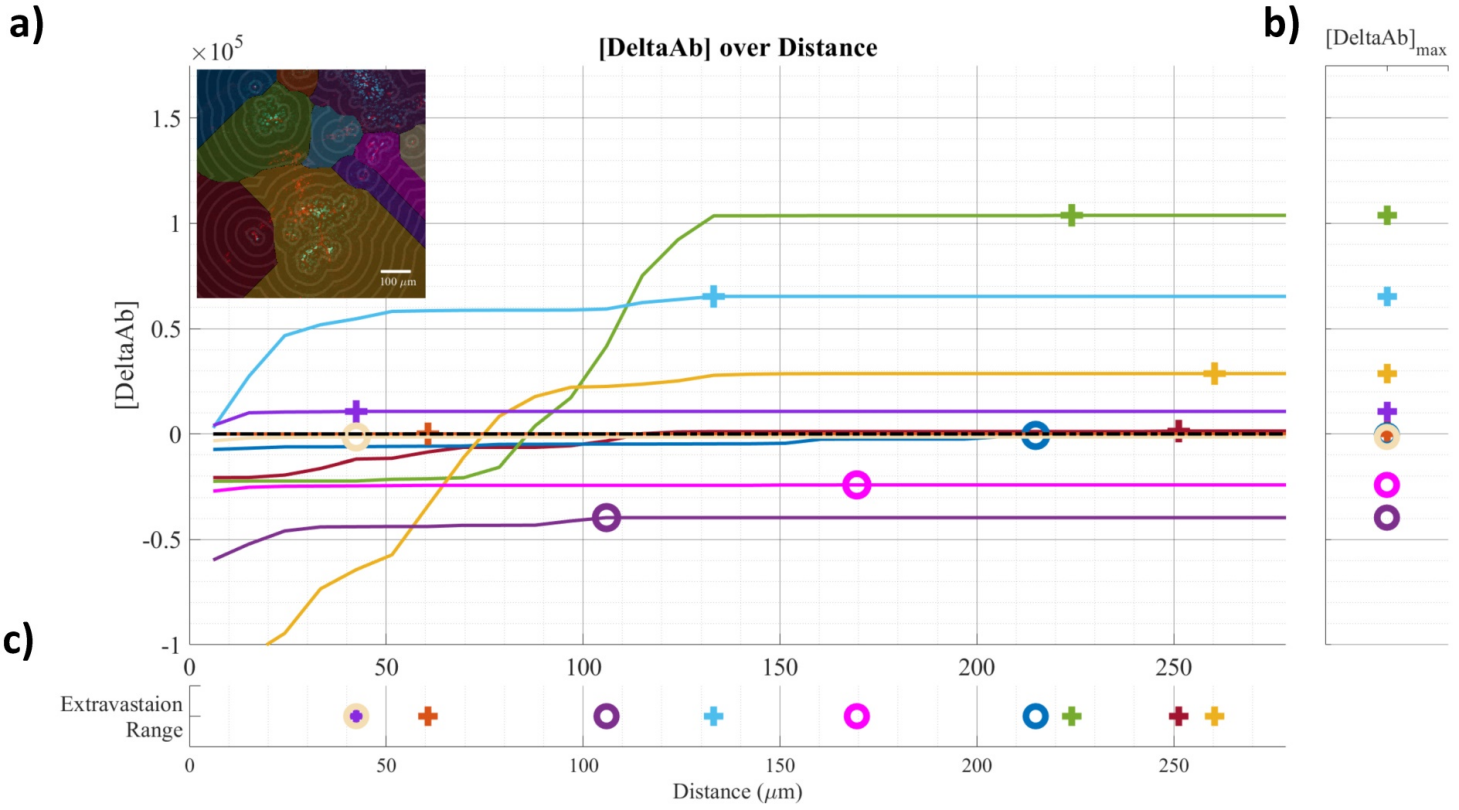
** Example of confocal data image quantification:** a) [DeltaAB] plotted as a function of distance for each vessel; b) [DeltaAB]_max_ is projected to the right for each vessel; c) the extravasation range is projected to the bottom for each vessel, defined as the location where [DeltaAB]_max_ reached its maximal value. Colors in a) correspond to the colored vessels in the upper left corner image, delimiting the area of influence of the vessel.

Frozen tumors were cryo-sectioned (5 μm), mounted (Prolong Gold, Invitrogen) and scanned using a confocal microscope (Leica TCS-SP5MP, 20x) to detect vessels (Hoechst) and antibodies (AF680) at respective (Ex/Em) wavelengths of (405 nm/460 nm) and (633 nm/682HP nm). The image processing is summarized in Figures 2-3. Briefly, a threshold was imposed on each channel using Otsu's method 22, which automatically selects a threshold by minimizing the variance within the two classes. Blue pixels in the resulting binary image located near each other were connected and defined as forming a vessel: this was obtained by sequentially filtering out noise (individual pixels) and performing two successive dilations: the first dilation (8 μm) was used to define the “inside” of the vessel; a second dilation (25 μm) was used to group closer agglomeration of cell nuclei into one continuous vessel. A watershed operation was done on the second dilated mask to obtain the area of influence of each vessel. The dilation parameters were identical for all the image analyses. A Euclidean distance transform (a map of the distance between each pixel and its nearest non- zero pixel 23) was applied to the 'inside' vessel mask to draw a series of masks delimiting increasing distances away from the closest vessel. For each vessel and within its watershed region, the integral of the antibody signal inside the vessel was subtracted from the integral of the antibody signal from outside the vessel to calculate the cumulated differential extravasated antibody signal (DeltaAb), defined as:


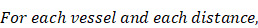



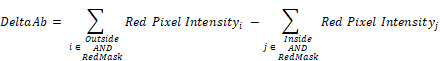


Where 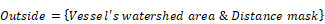



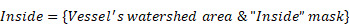


The area “outside” the vessel can be described as the pixels within a distance (d) in the watershed segmentation for the vessel but not inside the original “inside” mask. The red mask (a low-intensity threshold filter using Otsu's method) was necessary to eliminate integrating the red channel's noise. The extravasation range for each vessel was defined as the shortest distance away from the vessel where DeltaAb reached its maximum value, denoted [DeltaAb]_max_. (See Figure 3b). All vessels were thus classified as 'extravasated' ([DeltaAb]_max_>0) or 'not-extravasated' ([DeltaAb]_max_<0). A contingency table was created for all conditions and a Fisher's exact test was performed to compare the proportions of extravasated vessels with and without UTMC. Two-way ANOVA, followed by LSD Fisher post hoc multi-comparison tests, were used to compare [DeltaAb]_max_ and extravasation range. All calculations were done in MATLAB. Three tumors were analyzed for each group (excepted for the 500x10cyc group at 4 h, in which the Hoechst stain perfusion failed in one mouse) and over 90 vessels were analyzed in each group.

### Expression of CD73 in 4T1 cells

**Figure 4:**
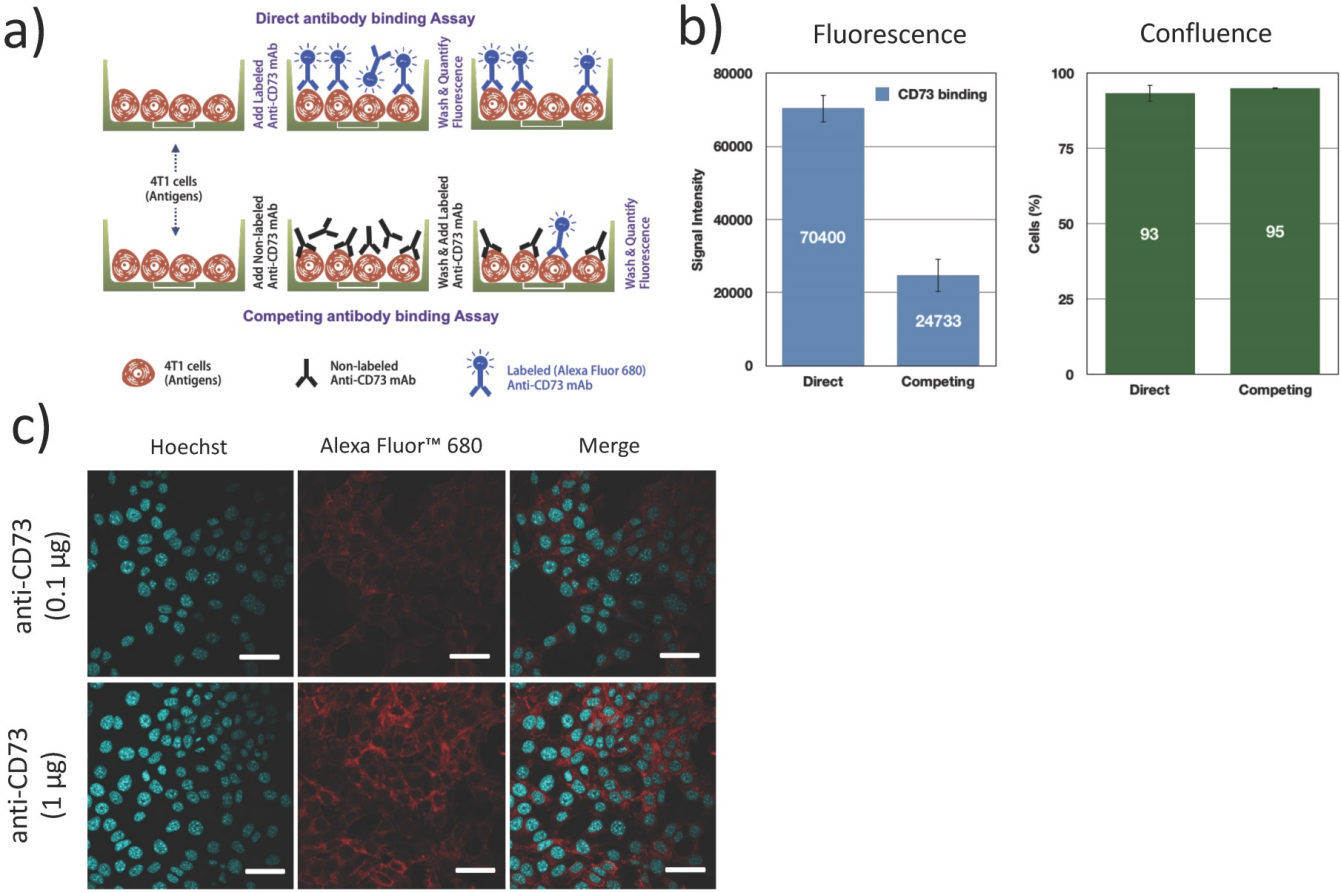
** Fluorescently labeled mAb (anti-CD73-F) binding to 4T1 cells:** (a) 4T1 cells were either exposed to fluorescently labeled antibodies (anti-CD73-F), or successively to anti-CD73 and anti-CD73-F in a competitive binding assay; (b) This competing assay with a prior native mAb challenge reduced the fluorescence/binding of anti-CD73-F, without changes in cell confluence (N=3); (c) Confocal microscopy supports that CD73 was expressed mostly on the membrane of 4T1 cells. Scalebar = 25μm.

The expression of CD73 on 4T1 cells and the functional binding of the fluorescently labeled antibodies were verified using a direct/competition ELISA assay. Briefly, 4T1 cells were cultured in 96 well plates, fixed (4% formaldehyde, 15 min), blocked (1% BSA + 0.1% Tween 20 in PBS, 1 h) and incubated with 100 μg/mL non-fluorescent anti-CD73 (competitive) antibodies or PBS (direct assay) for 1 h. After washing, cells were exposed to 1 μg/mL AF680 labeled anti-CD73 for 1 h and washed 3 times. Fluorescence was measured in an infrared imaging system (700 nm, Odyssey, LI-COR). All experiments were repeated 3 times. Confluence was visually inspected using bright field microscopy to ensure that cells did not detach during the washing and incubation steps (Figure 4b). Additionally, confocal microscopy was used to determine the localization of bound anti-CD73 antibodies. 4T1 cells were cultured on collagen (10 μg/cm^2^) coated coverslips blocked (1% BSA + 0.1% Tween 20 in PBS, 1 h), fixed (4% formaldehyde, 15 min) and incubated with anti-CD73-F antibodies (0.1 µg and 1 µg, 4 h at 4ºC) and Hoechst33342 (1 µg, 5 min, 20ºC). Cells were then imaged using confocal microscopy.

### Statistical analysis

All data were expressed as mean ± standard deviation unless specified. Statistical testing for biofluorescence was performed using two-way ANOVA to compare the effects of US pulse and treatment side (Figure 6b). Multiple comparisons analysis to discern differences between groups was performed using Sidak's post-hoc testing (Prism 8.0, Graphpad software, LaJolla, CA). For confocal microscopy, Fisher's exact test was used to analyze contingency tables and two-way ANOVA to compare the effects of US pulse and treatment side. Post-hoc LSD Fisher's multi-comparison testing was used to detect differences in [DeltaAb]_max_ and extravasation range (Prism 8.0, Graphpad software, LaJolla, CA).

## Results

### CD73 expression on 4T1 cells

We first confirmed CD73 expression on 4T1 murine breast tumor cells. Using a competitive/direct binding assay, we found that prior blocking of CD73 with unlabeled anti-CD73 mAb caused an important reduction (65%) in the fluorescence signal from bound anti-CD73-F mAb (Figure 4b), establishing that fluorescently labeled anti-CD73-F mAbs can specifically bind to 4T1 cells and conversely that 4T1 cells express CD73. Using confocal microscopy, we demonstrate that CD73 is highly expressed on 4T1 cell membrane, as expected 19.

### Bilateral model: Tumor growth data

**Figure 5:**
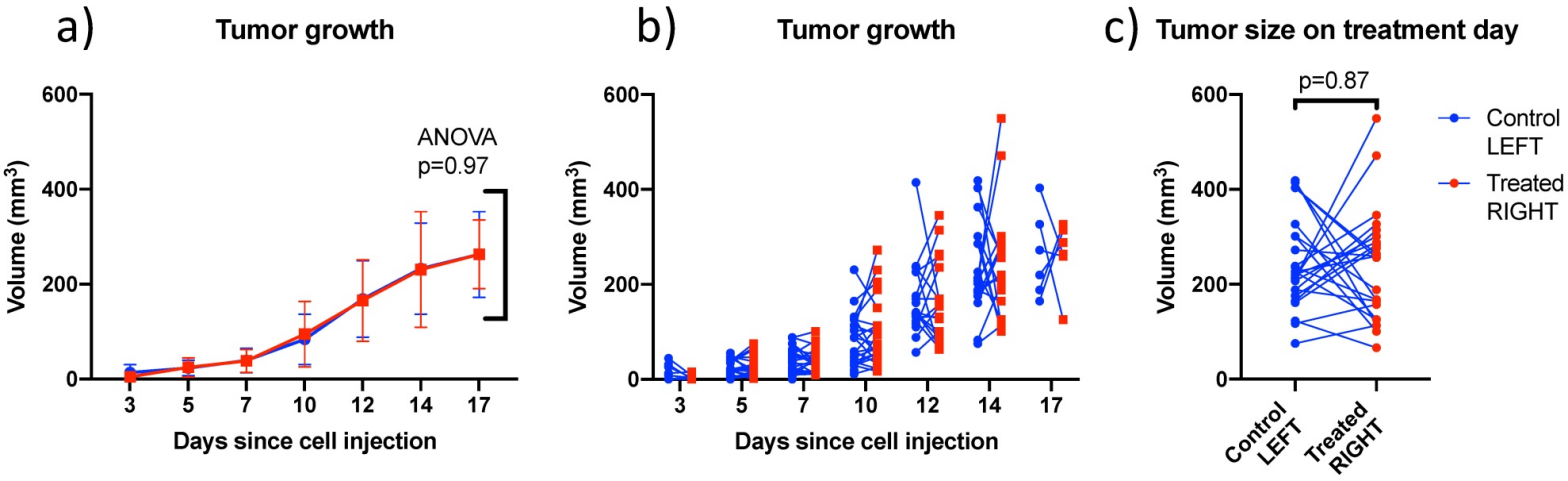
** Comparison of left and right tumor size over time and on treatment day:** (a) Mean bilateral tumor growth following 4T1 cell implantation in BALB/c mice; ANOVA did not detect a size difference as a function of side; (b) individual bilateral tumor sizes as a function of time showing that both sides were randomly larger or smaller; and (c) Tumor size on treatment day showing similar size on both sides on average. Lines join left and right tumors from the same mouse. (N=27)

4T1 tumors grew at similar rates in left and right sides (Figure 5) and were treated between days 12 and 17 when the individual tumor volumes reached a target size of 200-300 mm^3^. In this size range, tumors were vascularized but not necrotic based on CEUS imaging. There was no trend distinguishing the volumes of left (242 ± 96 mm^3^) and right (237 ± 118 mm^3^) tumors on the day of treatment. Tumors volumes ranged from 66 to 550 mm^3^ and were not larger on one particular side (Figure 5c).

### Biofluorescence increase on the treated side

**Figure 6:**
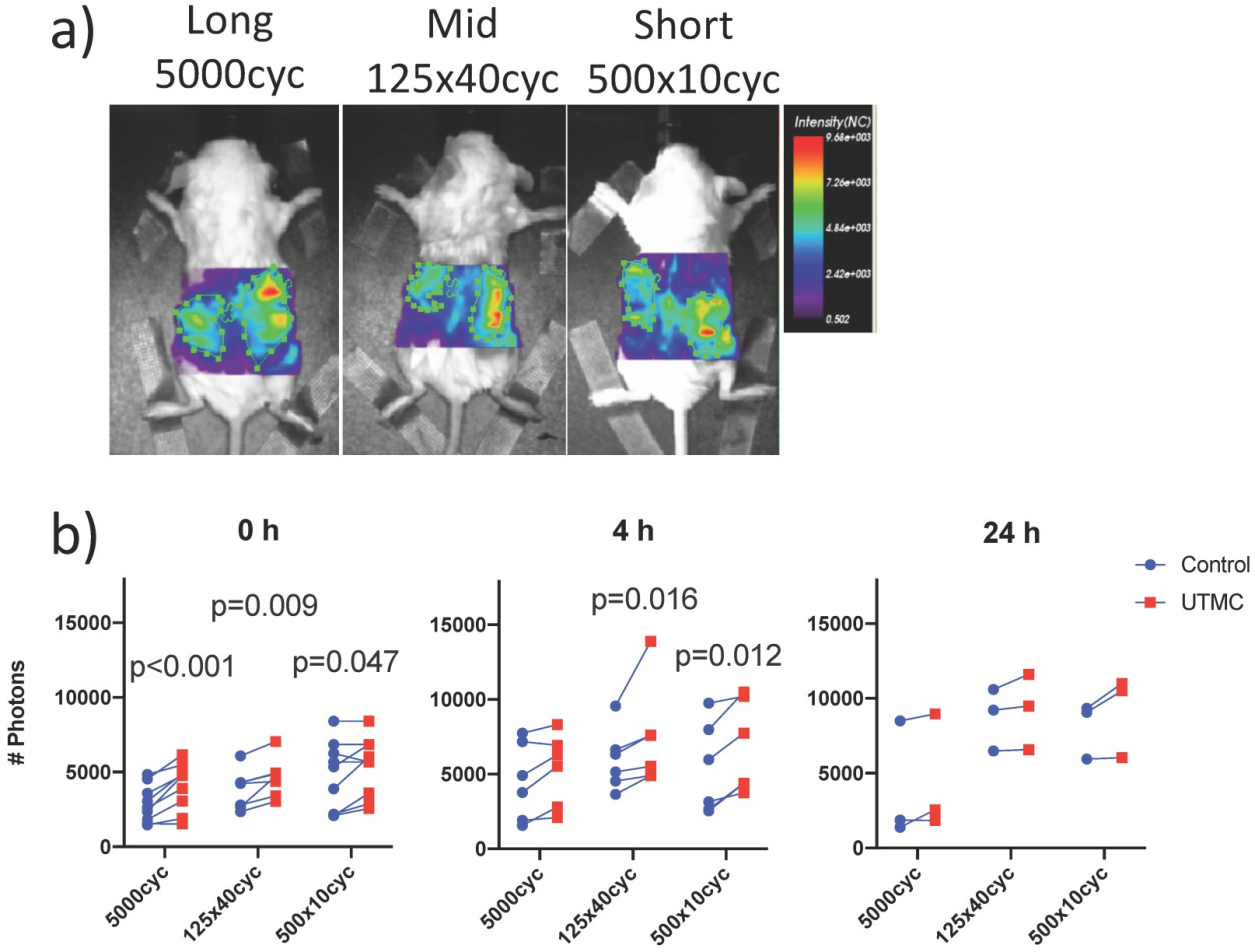
** Biofluorescence imaging:** a) Mice bearing bilateral tumors received an intravenous bolus of fluorescently labeled antibodies (anti-CD73-F) and were treated with UTMC on the right side only and were imaged by biofluorescence; b) Fluorescent signal for each mouse comparing right and left tumors at 0 h, 4 h and 24 h. Two-way ANOVA (side, pulse) was significant for side at all time points (p<0.02). Numerical p-values<0.05 are indicated (SIDAK multiple comparison testing. N=9 at 0 h, N=6 at 4 h and N=3 at 24 h.

UTMC treated and non-treated tumors pairs were imaged synchronously, such that all parameters besides US exposure (antibody injection dose, time in systemic circulation, gain and laser power of the bioimager) were the same for both tumors in every animal. Typical images are shown in Figure 6a. The fluorescence was higher on the treated side for almost every animal and at all time points (upward trending lines, Figure 6b), as supported by a strong effect of side (two-way ANOVA) at 0 h (p<0.0001), 4 h (p=0.0002) and a weaker effect of side at 24 h (p=0.021). Changing the pulse had no detectable effect at fixed time (two way ANOVA, all p>0.12). Upon multiple comparison testing, at 0 h, there was a difference between treated (right) and untreated (left) sides for all pulses (p<0.05): the largest difference was observed at for the long pulse (1184 photons, p=0.0002) and decreased for the mid and short pulses. At 4 h, this difference between right and left decreased for the long pulse (810 photons, p=0.23) but increased for the mid (1427 photons, p=0.01) and short (1453 photons, p=0.01) pulses. At 24 h, there were no differences between left and right upon multiple comparison testing (all p>0.09). The numerical values, pairwise comparisons and relative increase (normalized to control side at 0 h) are reported in Table 1. Note that there were lower numbers of observations with progressing time, as animals were sacrificed for histology, thus decreasing the power of the testing with increasing time.

### Biofluorescence increase with time

**Figure 7:**
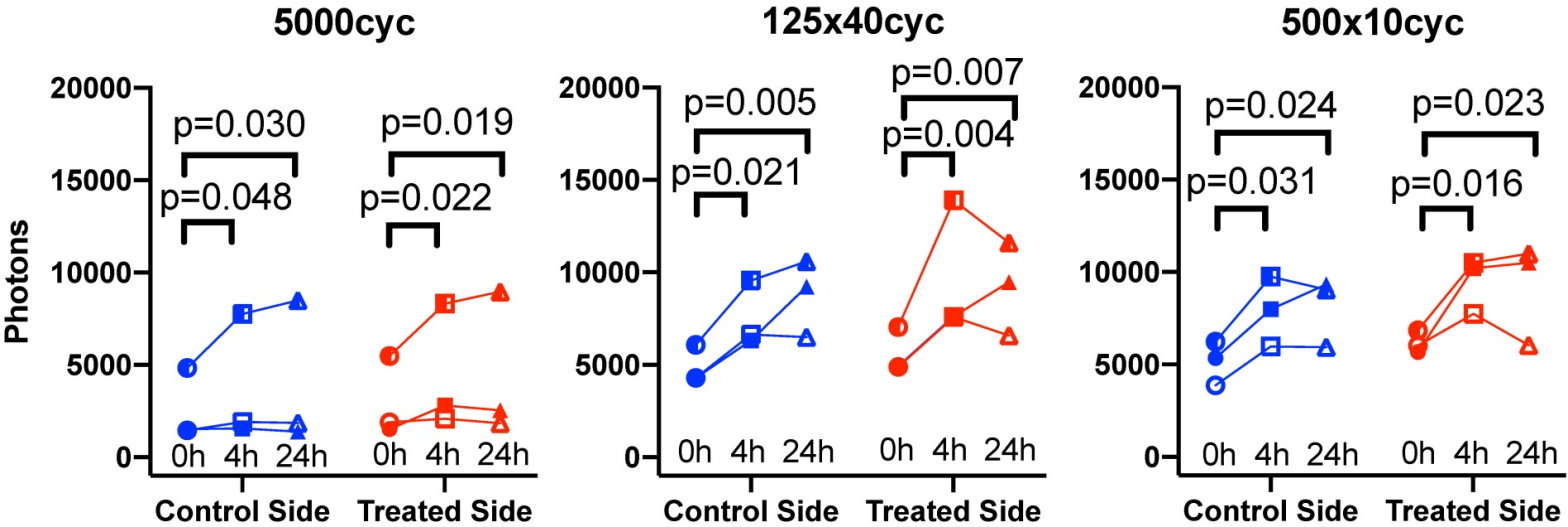
** Longitudinal biofluorescence signal as a function of side for different UTMC pulses:** The fluorescence signals measured in control and treated tumors are plotted as a function of time for the three mice per group that were serially imaged at 0 h, 4 h and 24 h. Data from the same animal are linked by a line. Compared to 0 h, the signal increased in all groups at 4 h and 24 h but was similar between 4 h and 24 h, indicating a plateau between 4 and 24 h. Numerical p-values<0.05 are indicated (SIDAK multiple comparison testing; N=3 per group).

To capture the effect of time, the data was replotted for the three animals that were imaged longitudinally at all timepoints for each pulse in Figure 7. Fluorescence increased with time: it was higher at 4 h (all p<0.05) and 24 h (all p<0.05) compared to 0 h, but was not different between 4 h and 24 h. Overall, this suggests an increase of antibody accumulation with time that plateaued between 4 h and 24 h. The % variation in fluorescence, normalized to the control side at 0 h, reported in Table 1, reflect the same plateau effect after 4 h.

### Confocal microscopy

**Figure 8:**
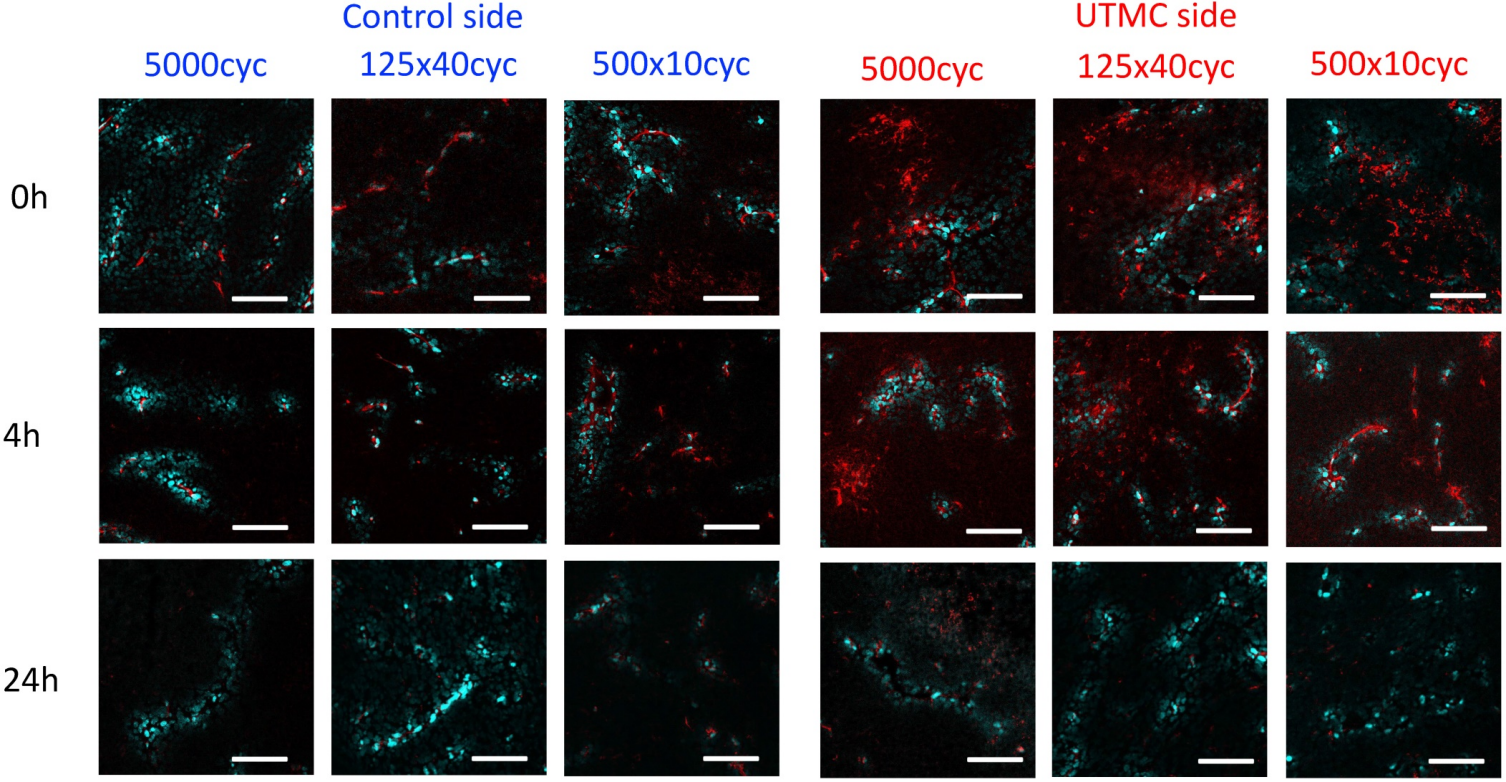
** The effect of UTMC on antibody microstructural distribution around vessels as a function of time on control and UTMC treated side for different ultrasound pulses:** Typical confocal images of tumors at 0 h, 4 h and 24 h after receiving UTMC. Vessels are stained in blue (perfusion stain injected IV before sacrifice) and antibodies carry a red fluorophore (AF680). Scale bar =100 μm.

Confocal microscopy was used to characterize the spatial distribution of the antibodies following UTMC. At 0 h and 4 h, antibodies appeared to be restricted to the vascular space in tumors that did not receive UTMC. Conversely, in tumors receiving UTMC, antibodies were found beyond the vascular space and penetrating into tumoral tissue. At 24 h the fluorescence signal was visibly weaker (Figure 8).

### Quantification of penetration in perivascular space

**Figure 9:**
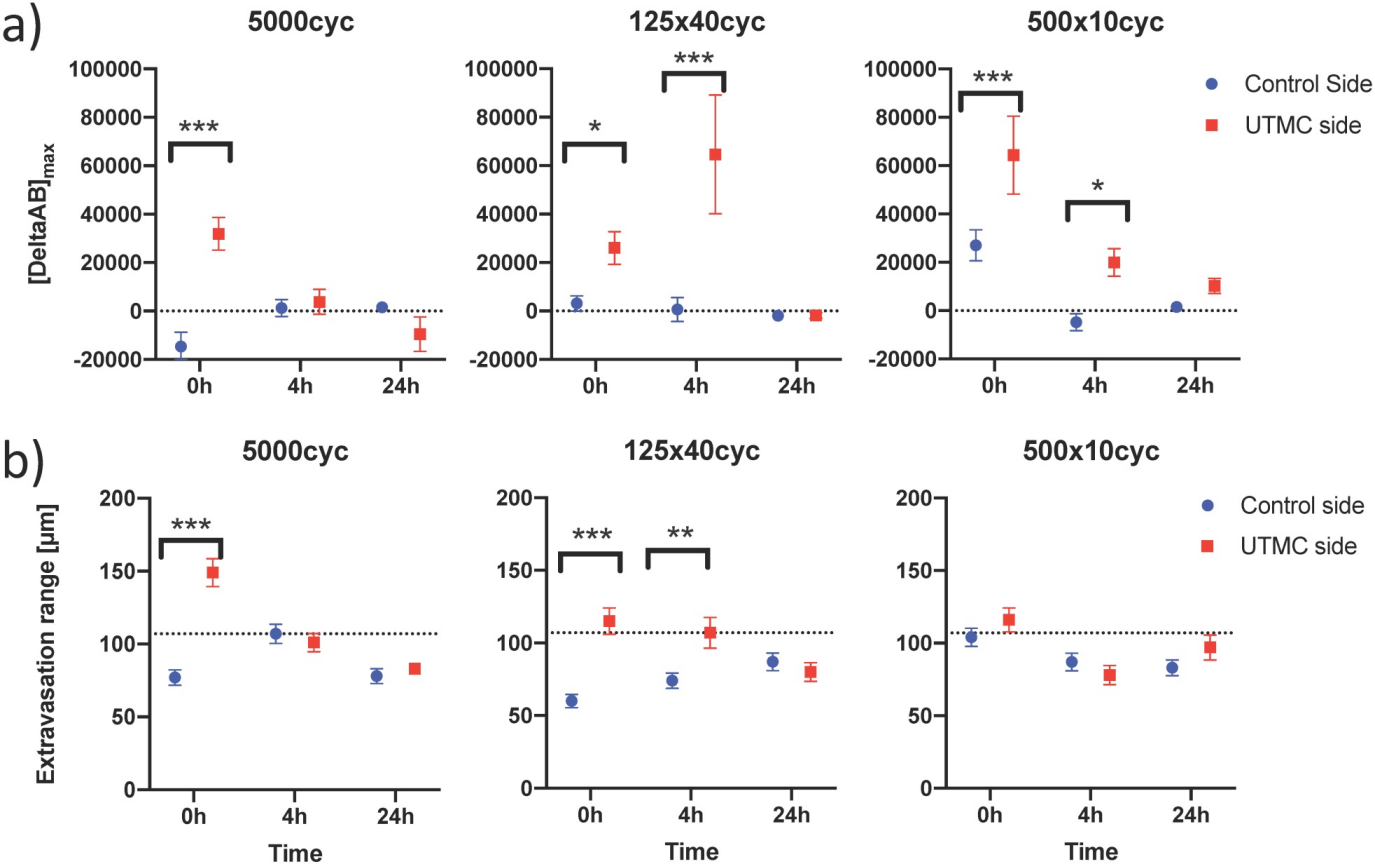
** Confocal data analysis**: (a) Differential extravasated antibody signal [DeltaAb]_max_; and (b) Extravasation range for each UTMC treatment as a function of side for each timepoint. Data in this graph are plotted as mean ± standard error of the mean. N=3 mice per group (only 2 mice for 500x10cyc at 4 h, see results section) and >90 vessels analyzed per side for each condition. Data was analysed using Two-way ANOVAs (side, time): for [DeltaAB]_max_, all p<0.05 for side and time; for extravasation range, all p<0.001 for side and time. *, ** and *** indicate respectively p<0.05, p<0.01 and p<0.001 from Fisher LSD multi comparison testing. Numerical data and statistics are reported in Table 3.

To characterize antibody extravasation, the sign of [DeltaAb]_max_ was used to group the vessels into 'extravasated' and 'not extravasated' categories. The counted vessels are reported in Table 2. We found that all three pulses caused an increase in the fraction of extravasated vessels in the treated tumors at 0 h and 4 h, which increased from a range of [35%-66%] (control side) to [60-96%] (UTMC side) (all p<0.03, Table 2). This increase in extravasated vessels was not found at 24 h (p>0.05). With the 5000cyc pulse, the ratio was even reversed at 24 h (more extravasated vessels in the control side) although the signal intensity was weak (see Figure 8 and Table 3). Similarly, we found that time and side had an effect on [DeltaAB]_max_ (two-way ANOVA, all p<0.05). [DeltaAB]_max_ increased for all UTMC pulses compared to control sides at 0 h (all p<0.05, Table 3), and for the mid and short pulses at 4 h (all p<0.03, Table 2), but was not different between treated and control sides at 24 h. Please find all the numerical values and pairwise comparisons statistics in Table 3. Finally, we found that time and side had an effect on the extravasation range (Two-way ANOVA, all p<0.001). The extravasation range was under 107 μm on the control side at all time points (Table 3) but increased above 107 um after UTMC (Figure 9 and Table 3): the extravasation range reached a maximum value of 149 μm for the long pulse at 0 h (p<0.001 vs control) which was higher than with the mid (115 μm, p=0.002) and short pulses (116 μm, p=0.003) at 0 h. However, the extravasation range with the long pulse was back to control values at 4 h and 24 h (respectively 101 and 83 μm, p>0.50). For the 125x40cyc group, the extravasation ranges were higher than control at 0 h (115 μm, p<0.001) and 4 h (107 μm, p=0.002), but were similar to control at 24 h (80 μm, p=0.42). For the 500x10 pulse, there was no difference in extravasation range at any timepoint. The summary of the comparisons and statistical tests can be found in Table 3.

## Discussion

### Ab tumoral accumulation following UTMC is dependent on the pulse length

In this study, we demonstrated that UTMC can increase the tumoral accumulation of anti-CD73 mAb in a syngeneic breast tumor model, at the macroscopic level, using whole body fluorescence, and at the microscopic level, using confocal microscopy. We used a bilateral tumor model so that each animal was its own control, in a longitudinal design with three timepoints (0 h, 4 h and 24 h). These time points were chosen based on the duration of BBB opening in the brain using UTMC, which is dependent on molecular size of the tracer, but typically begins to reverse at 6 h and is generally restored at 24 h 24-26. We found that there was a preferential accumulation of mAb in our model on the side receiving UTMC immediately after therapy (0 h timepoint), and that this differential increase persisted for at least 4 h after therapy, except for the 5000cyc pulse, consistently with blood brain barrier opening literature. Interestingly, despite treating tumors of heterogenous sizes (right bigger or smaller than left) (Figure 5b), the biofluorescence was systematically higher on the treated side at all timepoints (Figure 6b), a strong trend that was detected by two-way ANOVA testing (effect of side, all p<0.02). Upon multiple comparison testing, all the pulses had an effect on antibody accumulation at 0 h but only the mid and short pulses had an effect at 4 h, suggesting different accumulation kinetics with the pulse length (Figure 6b): the long pulse caused the highest antibody accumulation at 0 h on the treated side but this differential accumulation had vanished at 4 h; for the mid and short pulses, there was initially a smaller differential antibody accumulation at 0 h, which increased at 4 h but started to fade at 24 h (lower numerically and p>0.09 in multiple comparison testing). This plateauing at time > 4 h was also found in a subgroup of mice that were followed longitudinally at all time points (Figure 7), in which 4 h and 24 h data were not different. Finally, for the biofluorescence data, it is important to note that our study design intrinsically introduced a bias of decreasing statistical power with progressing time, because animals were sequentially sacrificed. This could in part explain why the difference in photons, which was positive for all pulses, but decreased with time (Table 1) could not be statistically detected. Overall, the biofluorescence data suggests that UTMC caused an increased accumulation of antibody in the treated tumor at 0 h and 4 h but this effect was not detected at 24 h post UTMC. Interestingly, these findings were corroborated at the microstructural level by confocal microscopy. Visually, it was clearly evident that UTMC caused the extravasation of fluorescent signal beyond the vascular space at 0 h and 4 h, whereas it remained mostly intravascular in the untreated tumors (Figure 8). This suggests that the increase in biofluorescence observed at the macroscopic level was caused by a modification at the microscopic level where antibodies could be found beyond the intravascular space and into the tumoral interstitium. We found that all three tested US pulses caused an increased proportion of vessels to allow antibody extravasation (defined as the difference between extravascular and intravascular antibody fluorescent signal) at 0 h and 4 h, but not at 24 h (Table 2). The amount of extravasated antibodies [DeltaAb]_max_ increased for all tested pulses at 0 h and for the mid and short pulse at 4 h, but not at 24 h. Finally, we also found that the extravasation range could be doubled with the longer pulses (5000cyc and 125x40cyc) at 0 h but that this increase persisted at the 4 h time point for the 125x40cyc pulse only. Interestingly, the short pulse (500x10cyc) did not cause an increase in extravasation range despite having a modest effect on [DeltaAb]_max_ and increasing the fraction of extravasated vessels. We could not detect differences at 24 h, at which time [DeltaAB]_max_ was very weak (Figure 8 and Table 3). Overall, based on Figures 6b, 7, 8 and 9 and Tables 1-3, our data supports that UTMC caused a targeted increased accumulation on the treated side that depended on the pulse length, in accordance with the biofluorescence data: for the long pulse, we found a stronger initial but short-lived UTMC mediated accumulation and longest extravasation range, which faded at the 4 h time point; for the mid and short pulses, there was an initial effect with lower [DeltaAB]_max_ and extravasation range, which persisted at 4 h but started to fade between 4 h and 24 h. Interestingly, the 125x40cyc pulse seemed to be a good middle ground, causing all metrics (biofluorescence, extravasated vessel fraction, [DeltaAB]_max_ and extravasation range) to increase at both 0 h and 4 h timepoints.

### Decrease of the extravasated signal with time

It was intriguing to find that the biofluorescence data was maintained at 24 h whereas the confocal extravasated data largely decreased at 24 h. This could be explained by the contribution of antibodies from the blood pool to the biofluorescence signal, whereas blood was flushed at sacrifice for the confocal data. This however does not explain why the extravasated signal decreased at 24 h in the confocal imaging data (e.g. the long range extravasated antibodies at 0 h with the long pulse disappeared with time) or explain the weaker antibody signal at 24 h on both sides in Figure 8. One possibility is the binding of the anti-CD73 mAb to its target, which can lead to the internalization and degradation of the antibody 27, 28. This anti-CD73 mAb (TY/23) has been used successfully in mice to target the CD73 receptor on 4T1 cells 5, 20, 21. Furthermore, although we did not experimentally confirm binding of the antibody to its CD73 receptor, it is unlikely that UTMC would affect the antibody function and binding ability for two reasons: (1) UTMC is applied only 5 minutes locally whereas antibodies are distributed systemically and accumulate over hours; (2) other antibodies have been used with UTMC and have demonstrated enhanced therapeutic efficacy which support that UTMC does not affect antibody binding 16, 17. Therefore binding of anti-CD73 mAbs to CD73 may lead to internalization and degradation of the antibody, although this was not demonstrated.

### Novelty

Thus, the findings of this study are in line with the well-known ability of UTMC to increase vascular permeability, for improving delivery of drugs, genes, nanoparticles, cells and antibodies to the brain 10-13, 29. For tumors, however, to the authors' knowledge, this is the first study describing the ability of UTMC to increase antibody accumulation and tumoral antibody extravasation *in vivo* outside the central nervous system and to provide detailed and simultaneous macroscopic and microstructural effects of UTMC therapy on antibody extravasation kinetics. Indeed, the distribution and tumoral penetration of antibodies in solid tumors is a major mechanism of resistance to antibody therapy 7, 30, 31 and thus enhancing and optimizing antibody penetration and accumulation could help enhance therapeutic efficacy. We found that UTMC allowed to increase the proportion of vessels presenting antibody extravasation and the extravasation upper range from 107μm to 149μm, which could help overcome the poor tumoral interstitial penetration of therapeutic compounds in solid tumors, and in particular bulky proteins like antibodies. More specifically, we provide the first evidence that we can enhance the delivery of a mAb targeting the CD73 pathway in 4T1 cells, which may open interesting therapeutic perspectives for increasing the efficacy of this particular immunotherapy strategy. Interestingly, while this approach was demonstrated here for anti-CD73 mAb, our theranostic approach could be extended to other antibody based therapies, including checkpoint blockade, to increase their efficacy or alternatively to reduce the dose and associated side effects and cost. Our results are also in line with the findings of Heath et al. 17, who demonstrated that UTMC enhanced the efficacy of cetuximab in a subcutaneous squamous cell carcinoma mouse model. It would be interesting to determine to what extent antibody tumoral penetration was achieved in the recent study by Goertz et al. 16, who recently demonstrated that UTMC enhanced the efficacy of anti-PD-1 therapy in a CT26 colorectal cancer model. It is important to note however that, contrarily to the approach used in their study in which higher pressures were used to cause tumoral vascular shutdown, our pulses did not cause vascular shutdown based on live tumor monitoring during treatment.

### Choice of US parameters

Vascular permeabilization typically increases with US pressure 14, 15, 32 but is limited by unwanted bioeffects, including hemorrhage 33 or sterile inflammation 34. While the BBB opening literature supports that limiting the pressure to non-inertial cavitation is indicated in order to minimize tissue damage, the paradigm is somewhat different for tumors, in which higher pressure, possibly causing increased vessel permeabilization at the cost of some tissue damage, is not a limiting factor. Indeed, higher pressure and/or MB cavitation can cause sterile inflammation in the brain, which could be a desirable bioeffect in the context of immunotherapy. For instance, high pressure UTMC (500 kHz, 100 ms duration, 1.4 MPa) caused an increase in heat shock protein expression and induced a T-cell immune response in a CT26 tumor model 14. Anti-PD-1 therapy in combination with high pressure UTMC causing vascular shut down (1 MHz, 50x100cyc, 1.65 MPa) was efficacious in a recent study combining UTMC and anti-PD-1 therapy 16. We thus decided to use the highest possible pressure available in our setup, namely 850 kPa. Interestingly, short and long pulses have been used in the BBB literature and we therefore designed our experimental conditions to explore different pulse lengths, but with the same total acoustical power and number of cycles. We were expecting differences since it is known that MB cavitation activity is higher with long pulses compared to shorter pulses 35-37. Within a very large parameter space for US pulse design, spanning from moderate (100μs) 16 to very long (2-10ms) pulse length in recent literature 15, 17, we opted for three pulses composed of the same total number of cycles but separated in trains of 1x 5000 cycles, 125 x 40 cycles and 500 x 10 cycles, with the objective of determining if pulse length played an role in UTMC antibody therapy.

### Limitations

There are a few limitations to the findings reported in this study. It is obvious that we only explored a very limited US parameter space and that further optimization will be needed to determine what the best UTMC parameters can cause the highest mAb extravasation. US pressure, frequency, treatment duration and the effects of MB size and concentration are a few parameters that could be optimized. Interestingly, the increased signal at 4 h and lack of signal at 24 h could be suggesting that UTMC should be repeated after the 4 h timepoint to further enhance the UTMC enhanced extravasation effects. Finally, this study results revealed macroscopic and microscopic evidence of tumoral antibody accumulation followed by UTMC are largely in line with other scientific reports and warrant further investigation to determine if UTMC can enhance antibody and immunotherapy efficacies. Finally, since we did not explore the therapeutic efficacy of this approach in this study, it remains unknown if the improved distribution following UTMC reported in this study will translate into an enhanced therapeutic efficacy. However, other groups have reported an enhancement in efficacy by combining UTMC with systemically injected antibodies such as anti-PD1 and anti-EGFR mAbs in rodent models. 16, 17. Our data supports that repeated UTMC treatment within the antibody circulation half-life, could improve the antibody accumulation in the treated area.

## Conclusion

In this study, we demonstrated that UTMC could enhance anti-CD73 mAb accumulation and tumoral distribution in a 4T1 mammary carcinoma model. The data presented in this study are robust, since macroscopic antibody accumulation, quantified by whole body biofluorescence imaging, was corroborated by microscopic confocal data, which demonstrated an increase in the amount of extravasated antibodies following UTMC on a pulse by pulse basis. The longest pulse (5000cyc) could double the extravasation range (up to 149 μm from the vessel wall) but this effect had faded 4 h post UTMC. Amongst the different pulse lengths tested, the 125x40cyc pulse was the most effective, since it was the only pulse increasing all three microscopic metrics, namely the fraction, amount and range of antibody extravasation at 0 h and 4 h time points. However, these effects were transient and had vanished at 24 h, indicating that repeated UTMC treatment after 4 h could enhance the observed effects. In this study, imaging was used both for guiding therapy and for longitudinal monitoring, which, along with a bilateral tumor design, allowed to achieve a tight control on the experimental conditions. We foresee that improving the distribution of anti-CD73 mAb could contribute to reduce adenosine and therefore stimulate the immune system against tumor cells and could help overcome the limited number of patients responsive to check point inhibition. Our data are in line with other studies supporting that UTMC can enhance antibody based therapies efficacy in solid tumors and may provide some insights on the mechanism by which this synergy can occur. Finally, while our study demonstrated the effects of UTMC on anti-CD73 mAb delivery in tumors, it is reasonable to think that our findings may be extended to other antibody-based therapies where ultrasound imaging is suitable.

## Figures and Tables

**Table 1:** Δ photons between UTMC-treated and non-treated sides and relative increase ratio normalized to the control side at t = 0 h, for each condition and time point. p<0.05 (Sidak multiple comparison testing) are highlighted in bold.

				Normalized fluorescence	
US pulse	Time	Δ photons	p-value	Control side	UTMC side
5000	0 h	1184 ± 739	<0.001	1	1.434 ± 0.308
	4 h	810 ± 772	0.229	1.453 ± 0.242	1.807 ± 0.352
	24 h	533 ± 593	0.507	1.313 ± 0.427	1.597 ± 0.307
125x40	0 h	808 ± 532	0.009	1	1.230 ± 0.218
	4 h	1427 ± 605	0.016	1.623 ± 0.219	1.955 ± 0.208
	24 h	450 ± 483	0.628	1.800 ± 0.324	1.883 ± 0.341
500x10	0 h	639 ± 897	0.047	1	1.223 ± 0.279
	4 h	1483 ± 800	0.012	1.422 ± 0.164	1.915 ± 0.170
	24 h	1067 ± 844	0.090	1.577 ± 0.155	1.767 ± 0.261

**Table 2:** Number of extravasated and not extravasated vessels found by confocal microscopy. p-values<0.05 are highlighted in bold.

		Control			Treated			Fisher's test
**US pulse**	**Time**	# ExV	# Not ExV	% ExV	# ExV	# Not ExV	% ExV	*p*-value
**5000**	0 h	**18**	**34**	**35**%	**34**	**4**	**89**%	**<0.001**
	4 h	**27**	**32**	**46**%	**46**	**18**	**72**%	**0.003**
	24 h	**49**	**14**	**78**%	**21**	**26**	**45**%	**<0.001**
**125x40**	0 h	**33**	**36**	**48**%	**43**	**9**	**83**%	**<0.001**
	4 h	**22**	**41**	**35**%	**27**	**18**	**60**%	**0.012**
	24 h	43	37	54%	28	34	45%	0.400
**500x10**	0 h	**38**	**20**	**66**%	**50**	**2**	**96**%	**<0.001**
	4 h	**20**	**19**	**51**%	**40**	**14**	**74**%	**0.029**
	24 h	40	37	52%	46	28	62%	0.250

ExV = Extravasated vessel, i.e. vessel with a positive [DeltaAB]_._

**Table 3:** Differential extravasation ([DeltaAB]_max_) and extravasation range between UTMC-treated and non-treated tumors, for each US pulse and time point. p<0.05 are highlighted in bold. Data expressed as mean ± standard error of the mean.

		[DeltaAB]_max_ (A.U.)			Extravasation Range (μm)		
US pulse	Time	Control	UTMC	Fisher LSD	Control	UTMC	Fisher LSD
**5000**	0 h	**-14612 ± 5848**	**31889 ± 6718**	**<0,001**	**77 ± 5**	**149 ± 10**	**<0.001**
	4 h	1210 ± 3502	3772 ± 5138	0.684	107 ± 7	101 ± 6	0.519
	24 h	1522 ± 425	-9509 ± 7100	0.114	78 ± 5	83 ± 4	0.615
**125x40**	0 h	**3101 ± 3139**	**26031 ± 6740**	**0.049**	**60 ± 5**	**115 ± 9**	**<0.001**
	4 h	**615 ± 4972**	**64647 ± 24500**	**<0.001**	**74 ± 5**	**107 ± 11**	**0.002**
	24 h	-1902 ± 1199	-1868 ± 1217	0.984	87 ± 6	80 ± 6	0.423
**500x10**	0 h	**27016 ± 6422**	**64377 ± 16119**	**<0.001**	104 ± 6	116 ± 8	0.223
	4 h	**-4737 ± 3501**	**19973 ± 5713**	**0.028**	87 ± 6	78 ± 7	0.403
	24 h	1518 ± 895	10284 ± 3104	0.314	83 ± 5	97 ± 9	0.096

## Abbreviations

mAb: monoclonal antibody
anti-CD73-F: Fluorescently labeled anti-CD73
AMP: adenosine monophosphate
ATP: adenosine triphosphate
BBB: blood brain barrier
BSA: bovine serum albumin
CEUS: contrast enhanced ultrasound
CHUM: Centre hospitalier de l'Université de Montréal
CRCHUM: CHUM research center
DeltaAb: cumulated differential antibody signal
[DeltaAb]_max_: maximum value of DeltaAb
MB: microbubble
PBS: phosphate buffered saline
Tregs: Regulatory T-cell
US: ultrasound
UTMC: ultrasound targeted microbubble cavitation

## References

[1] Scott AM, Wolchok JD, Old LJ (2012). Antibody therapy of cancer. Nat Rev Cancer.

[2] Chiavenna SM, Jaworski JP, Vendrell A (2017). State of the art in anti-cancer mAbs. J Biomed Sci.

[3] Sharma P, Allison JP (2015). The future of immune checkpoint therapy. Science.

[4] Allard B, Allard D, Buisseret L, Stagg J (2020). The adenosine pathway in immuno-oncology. Nat Rev Clin Oncol.

[5] Vijayan D, Young A, Teng MWL, Smyth MJ (2017). Targeting immunosuppressive adenosine in cancer. Nat Rev Cancer.

[6] Primeau AJ, Rendon A, Hedley D, Lilge L, Tannock IF (2005). The distribution of the anticancer drug Doxorubicin in relation to blood vessels in solid tumors. ClinCancer Res.

[7] Minchinton AI, Tannock IF (2006). Drug penetration in solid tumours. Nat Rev Cancer.

[8] Baker JHE, Kyle AH, Reinsberg SA, Moosvi F, Patrick HM, Cran J (2018). Heterogeneous distribution of trastuzumab in HER2-positive xenografts and metastases: role of the tumor microenvironment. Clin Exp Metastasis.

[9] Abrahao A, Meng Y, Llinas M, Huang Y, Hamani C, Mainprize T (2019). First-in-human trial of blood-brain barrier opening in amyotrophic lateral sclerosis using MR-guided focused ultrasound. Nat Commun.

[10] Burgess A, Hynynen K (2016). Microbubble-Assisted Ultrasound for Drug Delivery in the Brain and Central Nervous System. Adv Exp Med Biol.

[11] Alkins R, Burgess A, Ganguly M, Francia G, Kerbel R, Wels WS (2013). Focused ultrasound delivers targeted immune cells to metastatic brain tumors. Cancer Res.

[12] Burgess A, Hynynen K (2014). Drug delivery across the blood-brain barrier using focused ultrasound. Expert Opin Drug Deliv.

[13] Liu HL, Chen PY, Yang HW, Wu JS, Tseng IC, Ma YJ (2011). *In vivo* MR quantification of superparamagnetic iron oxide nanoparticle leakage during low-frequency-ultrasound-induced blood-brain barrier opening in swine. J Magn Reson Imaging.

[14] Liu HL, Hsieh HY, Lu LA, Kang CW, Wu MF, Lin CY (2012). Low-pressure pulsed focused ultrasound with microbubbles promotes an anticancer immunological response. J Transl Med.

[15] Yemane PT, Aslund AKO, Snipstad S, Bjorkoy A, Grendstad K, Berg S (2019). Effect of Ultrasound on the Vasculature and Extravasation of Nanoscale Particles Imaged in Real Time. Ultrasound Med Biol.

[16] Bulner S, Prodeus A, Gariepy J, Hynynen K, Goertz DE (2019). Enhancing Checkpoint Inhibitor Therapy with Ultrasound Stimulated Microbubbles. Ultrasound Med Biol.

[17] Heath CH, Sorace A, Knowles J, Rosenthal E, Hoyt K (2012). Microbubble therapy enhances anti-tumor properties of cisplatin and cetuximab *in vitro* and *in vivo*. Otolaryngol Head Neck Surg.

[18] Di Virgilio F, Sarti AC, Falzoni S, De Marchi E, Adinolfi E (2018). Extracellular ATP and P2 purinergic signalling in the tumour microenvironment. Nat Rev Cancer.

[19] Stagg J, Smyth MJ (2010). Extracellular adenosine triphosphate and adenosine in cancer. Oncogene.

[20] Whiteside TL (2017). Targeting adenosine in cancer immunotherapy: a review of recent progress. Expert Rev Anticancer Ther.

[21] Stagg J, Divisekera U, McLaughlin N, Sharkey J, Pommey S, Denoyer D (2010). Anti-CD73 antibody therapy inhibits breast tumor growth and metastasis. Proc Natl Acad Sci U S A.

[22] Otsu N (1979). A Threshold Selection Method from Gray-Level Histograms. IEEE Transactions on Systems.

[23] Maurer C, Qi R, Raghavan V (2003). A Linear Time Algorithm for Computing Exact Euclidean Distance Transforms of Binary Images in Arbitrary Dimensions. IEEE Transactions on Pattern Analysis and Machine Intelligence.

[24] O'Reilly MA, Hough O, Hynynen K (2017). Blood-Brain Barrier Closure Time After Controlled Ultrasound-Induced Opening Is Independent of Opening Volume. J Ultrasound Med.

[25] Mei J, Cheng Y, Song Y, Yang Y, Wang F, Liu Y (2009). Experimental study on targeted methotrexate delivery to the rabbit brain via magnetic resonance imaging-guided focused ultrasound. J Ultrasound Med.

[26] Choi JJ, Wang S, Tung YS, Morrison B 3rd, Konofagou EE (2010). Molecules of various pharmacologically-relevant sizes can cross the ultrasound-induced blood-brain barrier opening *in vivo*. Ultrasound Med Biol.

[27] Hay CM, Sult E, Huang Q, Mulgrew K, Fuhrmann SR, McGlinchey KA (2016). Targeting CD73 in the tumor microenvironment with MEDI9447. Oncoimmunology.

[28] Young A, Ngiow SF, Barkauskas DS, Sult E, Hay C, Blake SJ (2016). Co-inhibition of CD73 and A2AR Adenosine Signaling Improves Anti-tumor Immune Responses. Cancer Cell.

[29] Frank RT, Aboody KS, Najbauer J (2011). Strategies for enhancing antibody delivery to the brain. Biochim Biophys Acta.

[30] Cruz E, Kayser V (2019). Monoclonal antibody therapy of solid tumors: clinical limitations and novel strategies to enhance treatment efficacy. Biologics.

[31] Thurber GM, Schmidt MM, Wittrup KD (2008). Antibody tumor penetration: transport opposed by systemic and antigen-mediated clearance. Adv Drug Deliv Rev.

[32] Kinoshita M, McDannold N, Jolesz FA, Hynynen K (2006). Noninvasive localized delivery of Herceptin to the mouse brain by MRI-guided focused ultrasound-induced blood-brain barrier disruption. Proc Natl Acad Sci U S A.

[33] Price RJ, Skyba DM, Kaul S, Skalak TC (1998). Delivery of colloidal particles and red blood cells to tissue through microvessel ruptures created by targeted microbubble destruction with ultrasound. Circulation.

[34] Kovacs ZI, Burks SR, Frank JA (2018). Focused ultrasound with microbubbles induces sterile inflammatory response proportional to the blood brain barrier opening: Attention to experimental conditions. Theranostics.

[35] Chen X, Wang J, Pacella JJ, Villanueva FS (2016). Dynamic Behavior of Microbubbles during Long Ultrasound Tone-Burst Excitation: Mechanistic Insights into Ultrasound-Microbubble Mediated Therapeutics Using High-Speed Imaging and Cavitation Detection. Ultrasound Med Biol.

[36] Leeman JE, Kim JS, Yu FT, Chen X, Kim K, Wang J (2012). Effect of acoustic conditions on microbubble-mediated microvascular sonothrombolysis. Ultrasound Med Biol.

[37] Yu FTH, Chen X, Straub AC, Pacella J (2017). The role of nitric oxide during sonoreperfusion of microvascular obstruction. Theranostics.

